# The effect of Montmorency tart cherry consumption on athletic performance and post-exercise recovery in healthy adults: a scoping review

**DOI:** 10.3389/fnut.2026.1794292

**Published:** 2026-04-30

**Authors:** Yong Zhu, Jessica Smith, Amy Cohn

**Affiliations:** 1 Wayzek Science, St Paul, MN, United States; 2 Clarion Science, Long Valley, NJ, United States; 3 Cherry Marketing Institute, East Lansing, MI, United States

**Keywords:** athletic performance, Montmorency, muscle strength, post-exercise recovery, tart cherry

## Abstract

**Background:**

Various clinical studies have examined the effect of Montmorency tart cherry consumption on athletic performance and post-exercise recovery; however, there is a lack of a comprehensive review to assess the totality of evidence in the published literature.

**Methods:**

A scoping review was conducted to identify and summarize relevant clinical studies that examined the effect of Montmorency tart cherry consumption on athletic performance, post-exercise recovery of muscle strength, and delayed-onset muscle soreness (DOMS) in healthy adults.

**Results:**

A total of 28 published clinical trials met the eligibility criteria for the scoping review. Across these trials, heterogeneity was found in study design; however, the majority of studies had a small sample size with a short duration of intervention. Four out of 10 studies reported that Montmorency tart cherry consumption improved athletic performance with significantly shorter time to complete the exercise or longer time to exhaustion. Seven out of 14 studies reported a significant effect on post-exercise muscle strength recovery. Six out of 22 studies reported a significantly reduced DOMS with Montmorency tart cherry consumption.

**Conclusions:**

Overall, the evidence provides preliminary support for a potential beneficial effect of Montmorency tart cherry consumption on post-exercise recovery of muscle strength. Findings for athletic performance and DOMS are mixed, highlighting the need for further high-quality randomized controlled trials or systematic review with meta-analysis.

## 1 Introduction

High-intensity exercise may cause muscle damage due to oxidative stress, inflammation, or injury, resulting in temporary loss of muscle strength and the presence of muscle pain or soreness after exercise (1). Accumulating evidence suggests that supplementation with fruit high in polyphenols may have beneficial effects on athletic performance and post-exercise recovery (2, 3) potentially due to their anti-oxidative and anti-inflammatory properties. Among these, tart cherries (*prunus cerasus*) have received increased attention by both researchers and consumers.

Previous reviews have summarized the effect of tart cherry consumption on athletic performance or post-exercise recovery (4–8), which suggested tart cherry may boost exercise performance or improve post-exercise recovery. However, these reviews did not separate studies to assess outcomes for individual tart cherry varieties. In the United States, Amarelle and Morello are two common varieties of tart cherries and there are various cultivars within each variety; for example, Montmorency and Meteor are of the Amarelle variety whereas Northstar and Balaton are of the Morello variety (9). Moreover, modern breeding programs have created additional tart cherry cultivars. These different types of tart cherries may have different phytonutrient profiles. For instance, evaluation of 33 tart cherry cultivars revealed considerable variations in their nutritional and chemical compositions (10). Comparison of Montmorency with Balaton varieties also showed Montmorency tart cherries had 33% higher total phenolics, 341% higher melatonin, 181–421% higher flavonoids such as isorhamnetin rutinoside, kaempferol, and quercetin than Balaton tart cherries (11). These differences in phytonutrient compositions raised a question on whether potential functional benefit may differ by tart cherry varieties.

In the United States, 98% of tart cherries grown are the Montmorency variety (12). Despite being the dominating species of tart cherries in the United States, there is a lack of a review that specifically assesses the totality of evidence related to the role of Montmorency tart cherries in athletic performance and post-exercise recovery in healthy adults. Such assessment is critical to inform evidence-based recommendations for Montmorency tart cherries as functional foods and to guide the design of future studies. Therefore, the objective of this scoping review was to evaluate the effect of Montmorency tart cherries on athletic performance and post-exercise recovery, particularly with regard to muscle strength recovery and delayed-onset muscle soreness (DOMS) in healthy adults.

## 2 Methods

The scoping review was performed following the PRISMA Extension for Scoping Reviews (13).

### 2.1 Search strategy

A literature search was conducted in PubMed in May 2025 using search terms “sour cherry” OR “sour cherries” OR “tart cherry” OR “tart cherries” OR “Montmorency” OR “prunus cerasus”. To ensure that all relevant tart cherry interventions were captured, the search strategy included both general tart-cherry–related terms and the specific variety of interests in this review (Montmorency).

### 2.2 Eligibility criteria and study selection

Studies were included if they were published in English in a peer-reviewed scientific journal; were randomized controlled trials; included a healthy population; the intervention used a Montmorency tart cherry supplement, food, or juice that was not significantly altered nutritionally; included an appropriate control group that allowed for the isolation of the effect of tart cherries; and had direct measurements related to athletic performance (e.g., time to complete exercise, time to exhaustion, height of vertical jumped) or post-exercise recovery (e.g., muscle strength recovery and DOMS).

Studies were excluded if they were systematic, scoping, or narrative reviews; cell lines or animal studies; if there was a multi-component intervention that did not allow for the isolation of the impact of tart cherries; or if the study population was exclusively in a population that had a disease or other health condition, or if the tart cherry intervention used a variety/cultivar of tart cherry other than Montmorency. For studies that did not specify the variety/cultivar of tart cherry used, supplier information for the tart cherry was further assessed to verify Montmorency was the species used in the study.

Title/abstracts of the search results were manually screened by two reviewers, followed by full-text retrieval for relevant studies for further assessment. In addition, references from review papers were used as a potential resource for new studies. Studies that met all eligibility criteria as determined by two reviewers were retained for data extraction.

### 2.3 Data extraction

For each included study, the following information was extracted by two reviewers: authors, country of the study, year of publication; study design, trial registration; funding source; sample size, study population characteristics; tart cherry form, variety/species, source, dose and duration of Montmorency tart cherry intervention; duration of exercise pre-load period; duration of post-exercise follow-up; type of exercise, athletic performance or post-exercise recovery measures; and main results. In addition, whether the study was conducted in a laboratory or field setting was extracted. Quantitative quality and risk of bias assessments were not conducted due to the nature of a scoping review; however, notes were made for each study regarding clinical trial registration, identification of a primary outcome, population selection, and comments on study design, including identification of common methodological limitations. When the extracted data did not match, consensus was reached through discussions within the study team and by verification with information from the published studies.

### 2.4 Summarizing and reporting the results

One reviewer summarized the findings from the included studies and shared them with the study team for discussion and consensus. Results were reported separately for athletic performance (e.g., time to exhaustion, completion time for cycling or running), post-exercise muscle strength recovery (e.g., maximal voluntary contraction strength [MVCS] or peak torque), and DOMS.

## 3 Results

### 3.1 Characteristics of included studies

**Figure 1:**
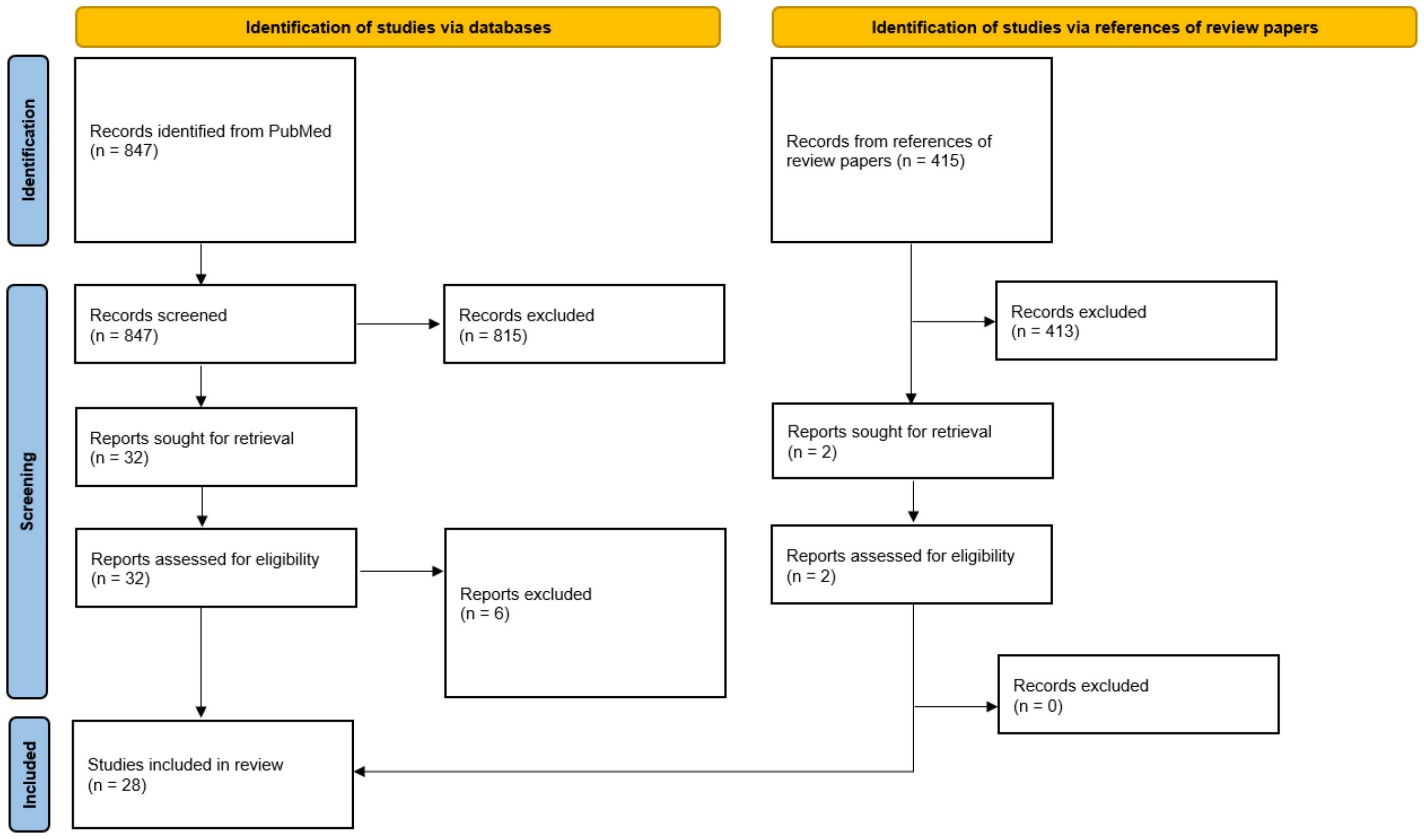
PRISMA flow diagram.

There were 847 papers from the initial PubMed search. Following screening of titles and abstracts, 32 studies were retrieved for full texts, and 26 studies met final study criteria. Two additional studies were added upon review of references from relevant review papers. After full text review, a total of 28 studies met final study criteria and were included in this scoping review (Figure 1).

Table 1 summarizes characteristics of the 28 studies included in this review. Most (18 out of 28) studies are cross-over trials. The total sample size varied from seven to 54 subjects. Nine studies included both men and women, whereas 17 studies included only male participants. The average age of participants in these studies varied between 18–38 years. Twelve studies were performed in subjects who were recreationally active or untrained, 15 studies involved professional or trained athletes, and one study included both recreationally active and trained adults. Six studies were field studies, in which participants completed a match or race, whereas the remaining studies were conducted in a laboratory setting.

**Table 1:** Characteristics of included studies.

Reference	Study design	Population	Sex	Age (y)	Dietary restriction	Tart cherry form	Company for tart cherry	Days of use	Exercise	Setting	Country of study	Trial registration	Funding source
Gao 2024 (14)	Cross-over trial	12 recreational cyclists	Both	35 ± 16	Yes	Juice	Prairie Fruit Processors Ltd, Canada	7	Cycling	Laboratory	Canada	Yes	Industry
Wangdi 2024 (16)	Cross-over trial	20 endurance-trained or recreationally active adults	Both	29 ± 6	Yes	Juice	Active Edge Ltd, UK	1	Steady-state exercise and cycling	Laboratory	UK and Australia	No	Academia
Horiuchi 2023 (17)	Cross-over trial	13 recreationally active adults	Both	21 ± 1	Yes	Capsule	Nature's Life, USA	5	Cycling	Laboratory	Japan	No	Other
Ortega 2023 (24)	Cross-over trial	17 recreationally active women	F	22 ± 3	Yes	Capsule	Toniiq LLC, USA	8	Concentric and eccentric muscle actions of the leg extensors	Laboratory	USA	No	No funding
Drummer 2022 (36)	Cross-over trial	Seven resistance-trained men	M	23 ± 4	No	Juice	CherryActive, Active Edge Ltd, UK	10	Unilateral resistance exercises	Laboratory	USA	No	Academia
Wangdi 2022 (25)	Cross-over trial	10 recreationally active men	M	23 ± 5	Yes	Juice	CherryActive, Active Edge Ltd, UK	10	Unilateral eccentric knee extension	Laboratory	UK and Australia	No	Industry
Hooper 2021 (37)	Cross-over trial	13 men with experience in barbell back squat	M	26 ± 5	No	Capsule	NordicCherry, Specnova, LLC, USA	7	Barbell back squat	Laboratory	USA	No	Industry
Abbott 2020 (38)	Cross-over trial	10 professional players	M	19 ± 1	No	Juice	Healthspan Ltd, UK	3	Soccer match	Field	UK	No	Not reported
Davis 2020 (18)	Cross-over trial	12 recreationally active men	M	28 ± 23	No	Capsule	Anderson Global Group, USA	7	Cycling	Laboratory	USA	No	Industry
Morehen 2020 (39)	Cross-over trial	11 professional players	M	18 ± 1	No	Juice	CherryActive, Active Edge Ltd, UK	7	Rugby league match	Field	UK	No	Not reported
Quinlan 2020 (26)	Parallel trial	20 adult team sport players	Both	26 ± 4	No	Juice	Holland and Barrett Ltd, UK	8	Adapted version of the Loughborough Intermittent Shuttle Test	Laboratory	UK	No	Not reported
Brown 2019 (27)	Parallel trial	20 physically active women	F	19 ± 1	No	Juice	CherryActive, Active Edge Ltd, UK	8	Repeated-sprint protocol	Laboratory	UK	No	Industry and academia
Kupusarevic 2019 (40)	Cross-over trial	10 elite players	M	28 ± 4	No	Juice	Healthspan Ltd, UK	5	Rugby union match	Field	UK	No	No funding
Lamb 2019 (28)	Parallel trial	36 untrained men	M	24 IQR (22–33)	Yes	Juice	CherryActive, Active Edge Ltd, UK	9	Eccentric exercise of the elbow flexors	Laboratory	UK	No	Academia
Morgan 2019 (19)	Cross-over trial	Eight trained cyclists	M	20 ± 2	No	Capsule	CherryActive, Active Edge Ltd, UK	7	Cycling	Laboratory	UK	No	Academia
Keane 2018 (20)	Cross-over trial	10 trained cyclists	M	28 ± 7	Yes	Juice	CherryActive, Active Edge Ltd, UK	1	Cycling and a 60-s all-out sprint	Laboratory	UK	No	Industry and academia
Beals 2017 (29)	Parallel trial	29 recreationally active adults	Both	18–50	No	Juice	TartVitaCherry, Futureceuticals, USA	12	Eccentric fatigue protocol of the quadriceps	Laboratory	USA	No	Industry
Bell 2016 (30)	Parallel trial	16 semi-professional players	M	25 ± 4	Yes	Juice	CherryActive, Active Edge Ltd, UK	8	Adapted version of the loughborough intermittent shuttle test	Laboratory	UK	No	Not reported
Levers 2016 (21)	Parallel trial	27 endurance-trained athletes	Both	22 ± 4	No	Capsule	CherryPURE, Shoreline Fruit LLC, USA	10	Half-marathon run	Field	USA	No	Industry
McCormick 2016 (15)	Cross-over trial	Nine trained players	M	19 ± 1	No	Juice	CherryActive, Active Edge Ltd, UK	6	Swimming-based tests	Laboratory	Australia	Yes	Other
Bell 2015 (32)	Parallel trial	16 trained cyclists	M	30 ± 8	Yes	Juice	CherryActive, Active Edge Ltd, UK	8	Cycling	Laboratory	UK	No	Industry
Levers 2015 (31)	Parallel trial	23 resistance-trained men	M	21 ± 3	No	Capsule	CherryPURE, Shoreline Fruit LLC, USA	10	Back squat exercise	Laboratory	USA	No	Industry
Clifford 2013 (22)	Cross-over trial	Nine trained athletes	M	32 ± 11	Yes	Capsule	CherryActive, Active Edge Ltd, UK	3	Cycling	Laboratory	UK	No	No funding
Kastello 2014 (33)	Cross-over trial	14 untrained adults	Both	21 ± 3	Yes	Tablet	CherryFlex, Brownwood Acre Foods Inc., USA	19	Eccentric arm extensions	Laboratory	USA	No	Industry
Bowtell 2011 (34)	Cross-over trial	10 well-trained athletes	M	28 ± 2	No	Juice	CherryActive, Active Edge Ltd, UK	10	Single-leg knee extensions	Laboratory	UK	No	Industry and other
Kuehl 2010 (41)	Parallel trial	54 healthy runners	Both	36 ± 10	No	Juice	Cherrish Inc., USA	8	Relay race	Field	USA	No	No funding
Howatson 2010 (23)	Parallel trial	20 recreational runners	Both	Cherry: 37 ± 13 Placebo: 38 ± 5	No	Juice	Cherrypharm Inc., USA	8	Marathon run	Field	UK	No	Academia
Connolly 2006 (35)	Cross-over trial	14 college students	M	22 ± 4	No	Juice	Cherrypharm Inc., USA	8	Eccentric elbow flexion contractions	Laboratory	USA	No	Industry

The majority of studies (19 out of 28) used Montmorency tart cherry juice (concentrated or unconcentrated); capsules or tablets of Montmorency tart cherry powder were used in nine studies. While the studies were conducted in United Kingdom, United States, Canada, Japan, or Australia, further research on the supplier information for Montmorency tart cherries revealed most studies used US-grown Montmorency tart cherries except one study used Canadian-grown Montmorency tart cherries (14).

The intervention period ranged from 1 to 19 days. The number of pre-load days varied from 0–16 days and the number of post-load days varied from 0–8 days. A variety of exercise modalities were assessed, with cycling being the most frequently studied. Only two studies registered their trials—one prospectively (14) and one retrospectively (15). Eleven studies implemented a dietary restriction protocol such as to limit or avoid consuming foods rich in polyphenols during the study. Thirteen studies reported receiving industry funding, whereas the remaining studies were supported by government, academic, or other sources, or reported no external funding.

### 3.2 Effect of Montmorency tart cherry on athletic performance

There were 10 studies that examined the effect of Montmorency tart cherry consumption on athletic performance (14–23) (Table 2). The doses (standardized to anthocyanins when applicable) varied from 66 to 2,760 mg/day and the supplementation period ranged from 1 to 10 days. Four of these 10 studies reported positive effects with Montmorency tart cherry consumption, such as improved time to completion of cycling tests (16, 19) or half-marathon running (21), or longer time to exhaustion in a cycling exercise (17). Notably, positive findings were observed in studies with recreationally active or untrained adults (17) as well as professional or trained athletes (19, 21), although *post-hoc* analysis from the study that included both recreationally active and trained adults showed a significant effect in trained adults but not untrained adults (16). The remaining studies reported no significant effect (14, 15, 18, 20, 22, 23).

**Table 2:** Randomized controlled trials that examined the effect of Montmorency tart cherry on athletic performance.

Reference	Study design	Population	Intervention	Exercise	Outcome	Treatment effect
Gao 2024 (14)	Cross-over trial	12 recreational cyclists, age 35 ± 16 years	Tart cherry juice (1,380 mg anthocyanin/150 ml) or placebo (high-glycemic index sport drink) consumed twice a day (300 ml/d) for 4 days before exercise and 2 days after exercise	A cycling protocol consisting of 90 min of cycling at 65% VO_2peak_ followed by a 10 km time trial	Time trial performance	No treatment effect. 17 ± 3 min for cherry juice vs. 17 ± 2 min for placebo, *P* = 0.27.
Wangdi 2024 (16)	Cross-over trial	20 endurance-trained or recreationally active adults, age 29 ± 6 years	Montmorency cherry concentrate (834 mg polyphenolics and 2,556 mg anthocyanins) administered as a single dose at 30, 90, or 150 min pre-exercise vs. an un-supplemented condition	A 10-min steady-state exercise and a 15-km cycling time trial	Time trial performance	Significant improvement in time to completion when Montmorency cherry concentrate was supplemented 90 min pre-exercise than un-supplemented condition (1,554.8 ± 226.7 s vs. 1,603.1 ± 248.0 s, *P* = 0.034). Analysis by training status showed a significant effect in trained but not recreationally active adults.
Horiuchi 2023 (17)	Cross-over trial	13 healthy young recreationally active adults, age 21 ± 1 years	Tart cherry capsule (100 mg anthocyanin) or placebo (flour) administered twice a day for 4 days, once on day 5 at 2 h before exercise	Incremental cycle exercise test to exhaustion in hypoxia conditions	Time to exhaustion	Time to exhaustion was significantly longer after tart cherry supplementation than placebo (940 ± 84 s vs. 912 ± 63 s, *P* = 0.01)
Davis 2020 (18)	Cross-over trial	12 recreationally active men, age 27.83 ± 22.62 years	500 mg of freeze-dried Montmorency cherry powder vs. placebo (cellulose) ingested daily for 7 days with exercise on day 7	Cycling at 70% of VO_2peak_ for a maximum of 30 min or until exhaustion.	Time to exhaustion and time to reach respiratory compensation point	No treatment effect. 21.46 ± 1.51 min for cherry powder vs. 19.50 ± 1.51 min for placebo, *P* = 0.36.
Morgan 2019 (19)	Cross-over trial	Eight trained male competitive cyclists, age 19.7± 1.6 years	Freeze-dried Montmorency cherry powder capsule (polyphenols 462.8 mg/d; anthocyanins 256.8 mg/d) vs. placebo (dextrose), six pills per day for 7 days; additional three pills taken 60 min before the experimental test on day 7	15 km cycling time trial	Completion time of a 15-km cycling time-trial.	The completion time was significantly faster following Montmorency cherry supplementation than placebo (1,506 ± 86 s vs. 1,580 ± 102 s, *P* < 0.01)
Keane 2018 (20)	Cross-over trial	10 trained male cyclists; age 28 ± 7 years	60 ml of Montmorency tart cherry concentrate or placebo (fruit-flavored cordial), diluted with 100 ml of water with exercise 1.5 h post-ingestion	Cycling test to exhaustion, and cycling test followed by a 60-s all-out sprint	Time to exhaustion	No treatment effect. 772 ± 34 s vs. 733 ± 34 s, *P* = 0.323.
Levers 2016 (21)	Parallel trial	27 endurance-trained runners or triathletes, age 21.8 ± 3.9 years	480 mg freeze-dried powdered Montmorency tart cherry supplement capsule (66 mg anthocyanin) or placebo (rice flour), taken once daily for 10 days, including the day of the race and up to 48 h post-run after breakfast	Half-marathon run	Half-marathon race split time and finish time	Both split time and finish time were significantly faster in subjects with supplement than placebo (49.03 ± 3.65 vs. 54.30 ± 4.18 min, *P* = 0.002; 103 ± 9.28 vs. 118 ± 9.72, *P* = 0.001)
McCormick 2016 (15)	Cross-over trial	Nine highly-trained male Water Polo players, age 18.6 ± 1.4 years	90 ml/d Montmorency tart cherry concentrate (9.117 mg/ml anthocyanin) diluted with water or placebo (fruit cordials) for 6 days (exercise on day 7)	The exercise protocol included swimming-based tests: in-water vertical jump test, 10 m sprint test, repeat sprint test, and Water Polo Intermittent Shuttle Test.	Performance outcomes include height in vertical jump, distance in Water Polo Intermittent Shuttle Test (WIST), and time for 10 m sprint test and repeat sprint test	No treatment effect. On day 7, vertical jump was 150 ± 6 vs. 150 ± 6 cm, distance in WIST was 605 ± 239 vs. 558 ± 203 m, 10 m sprint time was 5.59 ± 0.22 vs. 5.56 ± 0.15 s, for cherry juice vs. placebo.
Clifford 2013 (22)	Cross-over trial	Nine male cyclists or triathletes, age 32 ± 11 years	Montmorency tart cherry supplement (dried cherries with 216 mg of polyphenols) vs. another supplement (120 mg pycnogenol and 600 mg citrus bioflavonoids) vs. placebo (maltodextrin) for 2 days before and on the day of exercise	Cycling at four 5-min incremental stages followed by a 20 km cycling time trial	Time to complete trial	No treatment effect. Completion time was 2,008.56 ± 97.50 s for cherry supplement and 2,030.30 ± 124.73 s for placebo.
Howatson 2010 (23)	Parallel trial	20 recreational marathon runners, age 37 ± 13 years for tart cherry juice group and 38 ± 5 years for placebo group	Montmorency tart cherry juice (8 oz containing ≥600 mg phenolic compounds and ≥40 mg anthocyanins) or placebo (fruit-flavored water), twice a day, for 5 days before, the day of, and 48 h after a marathon run	A full marathon run	Marathon finish time	No treatment effect. Completion time (h:min:s) was 3:48:04 ± 0:48:58 for cherry juice vs. 4:15:48 ± 1:01:22 for placebo.

### 3.3 Effect of Montmorency tart cherry on post-exercise recovery of muscle strength

There were 14 studies that examined the effect of Montmorency tart cherry consumption on post-exercise recovery of muscle strength (14, 23–35) (Table 3). The doses (standardized to anthocyanins when applicable) varied from 15.4 to 2,760 mg/day and the supplementation period ranged from 7 to 19 days. Seven of these studies reported positive effects revealing a significantly higher maximum voluntary contraction force when supplemented with Montmorency tart cherries compared to a placebo (23, 25, 26, 30, 32, 34, 35). Of these seven studies with positive findings, four were performed in professional or trained athletes (26, 30, 32, 34).

**Table 3:** Randomized controlled trials that examined the effect of Montmorency tart cherry on post-exercise muscle strength recovery.

References	Study design	Population	Intervention	Exercise	Outcome	Treatment effect
Gao 2024 (14)	Cross-over trial	12 recreational cyclists, age 35 ± 16 years	Tart cherry juice (1,380 mg anthocyanin/150 ml) or placebo (high-glycemic index sport drink) consumed twice a day (300 ml/d) for 4 days before exercise and 2 days after exercise	A cycling protocol consisting of 90 min of cycling at 65% VO_2peak_ followed by a 10 km time trial	MVCS of knee extensors within 48 h of exercise	No treatment effect on MVCS over 48 h post-exercise
Ortega 2023 (24)	Cross-over trial	17 recreationally active women, age 22.2 ± 3.3 years	Tart cherry capsule (1,000 mg of concentrated tart cherry extract) or placebo (dextrose) daily for eight consecutive days, starting 4 days before the overload protocol, on the day of the protocol, and for 3 days after	Eight sets of 10 repetitions of maximal effort concentric and eccentric muscle actions of the leg extensors	Peak torque, time-to-peak torque within 3 days after exercise	No treatment effect on peak torque or time-to-peak torque over 3 days post-exercise
Wangdi 2022 (25)	Cross-over trial	10 recreationally active male participants, age 23.4 ± 5.4 years	Montmorency cherry concentrate supplementation (20.167 mg/ml polyphenolics and 7.211 mg/ml anthocyanin) vs. placebo (fruit cordial), two 30-ml doses per day for 10 days (7 days before exercise and 48 h after exercise)	Maximal unilateral eccentric knee extension trial	MVCS of knee within 48 h of exercise	Normalized maximum voluntary contraction 1-s average was significantly higher with supplementation than placebo (*P* = 0.024). *Post hoc* analysis showed a significantly higher force recovery for tart cherry immediately post-exercise (*P* = 0.033), but no significant differences at any other time point.
Quinlan 2020 (26)	Parallel trial	20 adult team sport players (football, hockey, netball), age 26 ± 4 years	Montmorency tart cherry concentrate (30 ml mixed with 70 ml of water) vs. placebo (fruit squash), twice per day (morning and evening), for eight consecutive days (5 days pre, day of, and 2 days post-Loughborough Intermittent Shuttle Test)	Adapted version of the Loughborough Intermittent Shuttle Test (LIST), consisting of 6 × 15 min sections from LIST Part-A, followed by 12 × 20 m maximal sprints with a 10 m deceleration zone, departing every 60 s.	Maximal voluntary isometric contraction (MVIC) of the non-dominant knee extensors within 48 h of exercise	Percent change in MVIC was significantly lower following supplementation than placebo at 24 and 48 h post-exercise (both *P* < 0.05; mean difference between groups was−38.4 and−49.8).
Brown 2019 (27)	Parallel trial	20 physically active females, age 19 ± 1 years	Montmorency tart cherry concentrate (30 ml of concentrate diluted in 100 ml of water, equivalent to 90 cherries) vs. placebo (fruit flavored concentrate), twice a day, for 8 days (4 days before exercise and 3 days after exercise)	A repeated-sprint protocol consisting of 15 × 30 m maximal sprints with a rapid 10 m deceleration phase, each separated by 60 s rest	MVCS of right knee extensors within 72 h of exercise	No treatment effect on MVCS over 72 h post-exercise
Lamb 2019 (28)	Parallel trial	36 non-resistance trained men, average age 24 years (IQR 22–33 years)	Tart cherry juice (30 ml concentrate with 220 ml water, 294.7 mg total phenolics and 7.7 mg anthocyanin) or Pomegranate juice (250 ml undiluted juice, 878.9 mg total phenolics and 49.4 mg	Eccentric exercise of the elbow flexors of the non-dominant arm, consisting of five sets of 10 repetitions of maximal voluntary eccentric contractions.	MVIC of elbow within 96 h of exercise	No treatment effect. The reduction in MIVC over 96 h post-exercise was fairly similar between groups (mean decrement was 14.6% for tart cherry and 17.3% for placebo).
			anthocyanin) or placebo (blackcurrant-flavored maltodextrin sports drink), twice a day for 9 days (exercise on day 5)			
Beals 2017 (29)	Parallel trial	29 recreationally active adults aged 18–50 years	60 g freeze-dried tart cherry powder (0.5% anthocyanin) mixed with 40 oz fluid, or placebo drink (black cherry Kool-Aid mixed with rice protein powder), twice a day, for 12 days (4 days before and 7 days after the fatigue protocol)	An eccentric fatigue protocol involving five maximal effort isokinetic concentric/eccentric trials of the quadriceps at 60 degrees per second	Isokinetic strength of the quadriceps within 1 week of the fatigue protocol	No treatment effect. There was also no significant change over time in both groups.
Bell 2016 (30)	Parallel trial	16 semi-professional male soccer players, age 25 ± 4 years	Montmorency tart cherry concentrate (30 ml, 73.5 mg/L cyanidin-3-glucoside) vs. placebo (fruit cordial with water and maltodextrin), twice a day for 8 consecutive days (exercise on day 5)	Adapted version of the Loughborough Intermittent Shuttle Test (LIST), with a series of 12 × 20-meter sprints with a 10-meter stopping zone, departing every 60 seconds, and 6 × 15-min sections from the LIST Part A.	MVIC of knee extensors within 72 h of exercise	Percent change in MVIC was significantly lower following supplementation than placebo (*P* = 0.001). The decline was not evidence in tart cherry group but it did not return to baseline levels at 72 h in the placebo group.
Levers 2015 (31)	Parallel trial	23 resistance-trained males, age 20.9 ± 2.6 years	480 mg freeze-dried powdered Montmorency tart cherry supplement capsule (40 mg anthocyanin per 290 mg) or placebo (rice flour), taken once daily for 10 days (7 days before exercise, on the day of exercise, and for 2 days after exercise)	10 sets of 10 repetitions at 70% of 1-RM back squat exercise	MVCS using an isokinetic knee extension/flexion test within 48 h of exercise	No treatment effect in both MVCS in knee extension/flexion total work performance over 48 h post-exercise.
Bell 2015 (32)	Parallel trial	16 trained male cyclists, age 30 ± 8 years	Montmorency cherry concentrate (30 ml with 9.2 mg/ml anthocyanin) or placebo (mixed berry cordial), mixed with 100 ml water, twice a day, for 8 consecutive days (4 days pre-trial, on the day of, and 3 days post-trial)	A 109-min cycling trial designed to replicate road race demands	MVCS was measured using a strain gauge attached to the dominant ankle within 72 h of exercise	MVCS decline was significantly attenuated in the supplemented group compared to the placebo group (*P* = 0.014), between-group difference was 10, 12 and 21% at 24, 48 and 72 h.
Kastello 2014 (33)	Cross-over trial	14 untrained adults, age: 21.3 ± 2.8 years	Tart cherry supplement with other ingredients (containing 100 mg anthocyanins, 20 mg flavones, and 30 mg tannins, melatonin, and two flavonoids isoquercitrin and quercitrin) or placebo (cooking oil with food color), taken twice a day for 16 days prior to and 3 days following eccentric exercise protocol	Five sets of 10 maximal eccentric arm extensions	Percent peak torque loss following maximal eccentric arm contractions within 3 days after exercise	No treatment effect on percent of peak torque loss over 72 h post-exercise
Bowtell 2011 (34)	Cross-over trial	10 well-trained male athletes participating in high-intensity intermittent sports with regular resistance training, age 27.8 ± 1.6 years	30 ml of Montmorency cherry juice concentrate (9.117 mg/ml anthocyanin) or placebo (synthetically derived fruit concentrate) twice per day for 10 days (7 days before exercise and 48 h after exercise)	10 sets of 10 single-leg knee extensions at 80% one-repetition maximum	Knee extension MVCS was measured within 48 h of exercise	MVCS recovery was significantly faster when supplemented than placebo (*P* = 0.04 for treatment by time interaction); levels returning to 90.9% for tart cherry vs. 84.9% for placebo after 24 h, and 92.9 vs. 88.5% after 48 h.
Howatson 2010 (23)	Parallel trial	20 recreational marathon runners, age 37 ± 13 for tart cherry juice group and 38 ± 5 for placebo group	Montmorency tart cherry juice (8 oz containing ≥600 mg phenolic compounds and ≥40 mg anthocyanins) or placebo (fruit-flavored water), twice a day, for 5 days before, the day of, and 48 h after a marathon run	A full marathon run	MVCS was measured using a strain gauge attached to the non-dominant ankle within 48 h of exercise	Significant greater recovery of maximum voluntary contraction strength in the supplemented group compared to the placebo group over 48 h (*P* = 0.024).
Connolly 2006 (35)	Cross-over trial	14 male college students, age 22 ± 4 years	12 fl oz of a Montmorency tart cherry juice blend (containing at least 600 mg phenolic compounds and at least 40 mg anthocyanins) or placebo (black berry Kool-Aid drink with water), twice a day for eight consecutive days (exercise on day 4)	A bout of eccentric elbow flexion contraction (2 by 20 maximum contractions)	Isometric elbow flexion strength within 4 days after exercise	Cherry juice resulted in significant less loss in isometric elbow flexion strength than placebo (treatment by time interaction *P* < 0.0001); pairwise comparison showed significantly less loss with tart cherry at 24, 48, 72, and 96 h after Exercise.

Eleven of the 14 studies also measured blood creatine kinase activities as a biomarker for muscle damage (23, 25–34); none of them reported a significant treatment effect on creatine kinase activities (data not shown).

### 3.4 Effect of Montmorency tart cherry on delayed-onset muscle soreness

There were 22 studies that examined the effect of Montmorency tart cherry consumption on DOMS (14, 15, 21, 23–41) (Table 4). Most studies used subjective measures such as visual analog scales, although some incorporated objective assessments using algometers (Table 4). The doses (standardized to anthocyanins when applicable) varied from 15.4 to 2,760 mg/day and the supplementation period ranged from 3 to 19 days. Six of the 22 studies reported significantly lower DOMS following Montmorency tart cherry supplementation compared with placebo (21, 30, 31, 33, 35, 41); of these studies, three were studies with professional or trained athletes (21, 30, 31), while the remainder did not observe significant differences.

**Table 4:** Randomized controlled trials that examined the effect of Montmorency tart cherry on delayed-onset muscle soreness.

Reference	Study design	Population	Intervention	Exercise	Soreness measures	Treatment effect
Gao 2024 (14)	Cross-over trial	12 recreational cyclists, age 35 ± 16 years	Tart cherry juice (1,380 mg anthocyanin/150 ml) or placebo (high-glycemic index sport drink) consumed twice a day (300 ml/d) for 4 days before exercise and 2 days after exercise	A cycling protocol consisting of 90 min of cycling at 65% VO_2peak_ followed by a 10 km time trial	Algometer and VAS	No treatment effect on muscle soreness over 48 h post-exercise (*P* > 0.05).
Ortega 2023 (24)	Cross-over trial	17 recreationally active women, age 22.2 ± 3.3 years	Tart cherry capsule (1,000 mg of concentrated tart cherry extract) or placebo (dextrose) daily for eight consecutive days, starting 4 days before the overload protocol, on the day of the protocol, and for 3 days after	Eight sets of 10 repetitions of maximal effort concentric and eccentric muscle actions of the leg extensors	VAS	No treatment effect on muscle soreness over 72 h post-exercise (*P* = 0.874).
Drummer 2022 (36)	Cross-over trial	Seven resistance-trained males, age 22.9 ± 4.1 years	Montmorency Cherry Juice (30 ml, 320 mg anthocyanin) concentrate or placebo (Kool-Aid drink), twice a day for 10 days (exercise on day 7)	Unilateral resistance exercises with single-leg goblet step-up, single-leg extension, single-leg curl, single-arm dumbbell press, and single-arm dumbbell row. four working sets with 3 RM for goblet step-up and 6 RM for others.	VAS	No treatment effect on change in muscle soreness over 72 h post-exercise (*P* = 0.28).
Wangdi 2022 (25)	Cross-over trial	10 recreationally active male participants, age 23.4 ± 5.4 years	Montmorency cherry concentrate supplementation (20.167 mg/ml polyphenolics and 7.211 mg/ml anthocyanin) vs. placebo (fruit cordial), two 30-ml doses per day for 10 days (7 days before exercise and 48 h after exercise)	Maximal unilateral eccentric knee extension trial	VAS and algometer	No treatment effect on soreness measure (*P* = 0.481) or pain pressure threshold (*P* = 0.963) over 48 h post-exercise; at 48 h, mean soreness from VAS was 44 vs. 43 mm for tart cherry vs. placebo; pain pressure threshold sum from three muscles was 129.6 vs. 119.5 N for tart cherry vs. placebo.
Hooper 2021 (37)	Cross-over trial	13 men with prior experience in the barbell back squat, age 26.2 ± 5.3 years	500 mg of powdered tart cherry extract capsule (5–6% polyphenols) vs. placebo (rice flour) daily for 7 days (exercise on day 7)	Resistance exercise protocol consisting of six sets of 10 repetitions of barbell back squat with 80% 1 RM.	VAS	No treatment effect on muscle soreness over 48 h post-exercise (*P* = 0.136)
Abbott 2020 (38)	Cross-over trial	10 male professional soccer players, age 19 ± 1 years	Tart cherry juice (30 ml concentrate, equivalent to 100 sour cherries) vs. placebo (cherry-flavored control drink) before and after the match, and 12 and 36 h after the match	A 90-min competitive soccer match	VAS	No treatment effect on muscle soreness over 60 h post-match (*P* = 0.808)
Morehen 2020 (39)	Cross-over trial	11 male professional rugby league match players, age 18 ± 1 years	Montmorency cherry juice (320 mg anthocyanin per 30 ml) vs. placebo (fruit cordial), consumed twice daily for 7 consecutive days (5 days pre-match, match day, and 2 days post-match)	Professional Rugby League match-play	Likert scale	No treatment effect on muscle soreness at 24 or 48 h post-match (*P* > 0.05)
Quinlan 2020 (26)	Parallel trial	20 adult team sport players (football, hockey, netball), age 26 ± 4 years	Montmorency tart cherry concentrate (30 ml mixed with 70 ml of water) vs. placebo (fruit squash), twice per day (morning and evening), for eight consecutive days (5 days pre, day of, and 2 days post-Loughborough Intermittent Shuttle Test)	Adapted version of the Loughborough Intermittent Shuttle Test (LIST), consisting of 6 × 15 min sections from LIST Part-A, followed by 12 × 20 m maximal sprints with a 10 m deceleration zone, departing every 60 s.	VAS	No treatment effect on muscle soreness over 48 h post-exercise (*P* = 0.262). A significant treatment by time interaction was found (*P* = 0.014) but *post-hoc* analysis failed to identify any pair-wise comparison that was significantly different.
Brown 2019 (27)	Parallel trial	20 physically active females, age 19 ± 1 years	Montmorency tart cherry concentrate (30 ml of concentrate diluted in 100 ml of water, equivalent to 90 cherries) vs. placebo (fruit flavored concentrate), twice a day, for 8 days (4 days before exercise and 3 days after exercise)	A repeated-sprint protocol consisting of 15 × 30 m maximal sprints with a rapid 10 m deceleration phase.	VAS and algometer	No significant treatment effect on muscle soreness although there was a trend toward lower DOMS in the tart cherry group vs. placebo (*P* = 0.070). There was a trend toward a higher pain pressure threshold in the tart cherry group vs. placebo (*P* = 0.071).
Kupusarevic 2019 (40)	Cross-over trial	10 elite male rugby union players, age 28 ± 4 years	Sour Montmorency tart cherry juice (30 ml) or placebo (food gel), twice daily for 5 days (2 days before the match, the day of the match, and 2 days after the match)	An 80-min rugby union match.	VAS	No treatment effect on muscle soreness at any time point (1, 2, 3 days after match, *P* = 0.807)
Lamb 2019 (28)	Parallel trial	36 non-resistance trained men, average age 24 years (IQR 22–33 years)	Tart cherry juice (30 ml concentrate with 220 ml water, 294.7 mg total phenolics and 7.7 mg anthocyanin) or Pomegranate juice (250 ml undiluted juice, 878.9 mg total phenolics and 49.4 mg anthocyanin) or placebo (blackcurrant-flavored maltodextrin sports drink), twice a day for 9 days, exercise on day 5	Eccentric exercise of the elbow flexors of the non-dominant arm, consisting of 50 maximal voluntary eccentric contractions	VAS	No treatment effect on elbow flexor soreness (*P* = 0.32); mean increase in soreness was 17.2 mm in the tart cherry group and 11.7 mm in the placebo group.
Beals 2017 (29)	Parallel trial	29 recreationally active adults aged 18–50 years	60 g freeze-dried tart cherry powder (0.5% anthocyanin) mixed with 40 oz fluid, or placebo drink (black cherry Kool-Aid mixed with rice protein powder), twice a day, for 12 days (4 days before and 7 days after the fatigue protocol)	Eccentric fatigue protocol involving repetitive, maximal effort isokinetic concentric/eccentric contractions of the quadriceps	VAS	No treatment effect on muscle soreness at 24, 48, 96 h or 1 week post-exercise (*P* > 0.05)
Bell 2016 (30)	Parallel trial	16 semi-professional male soccer players, age 25 ± 4 years	Montmorency tart cherry concentrate (30 ml, 73.5 mg/L cyanidin-3-glucoside) vs. placebo (fruit cordial with water and maltodextrin), twice a day for 8 consecutive days (exercise on day 5)	Adapted version of the Loughborough Intermittent Shuttle Test (LIST), which involved a series of 12 20 m sprints with a 10 m stopping zone, departing every 60 seconds, and six 15 min sections from the LIST Part A.	VAS	DOMS ratings were significantly lower in the tart cherry group compared to the placebo group at 24, 48, and 72 h post-exercise (60 vs. 93 mm, 44 vs. 90 mm, 10 vs. 33 mm; *P* = 0.044, *P* = 0.018, *P* = 0.007, respectively).
Levers 2016 (21)	Parallel trial	27 endurance-trained runners or triathletes, age 21.8 ± 3.9 years	480 mg freeze-dried powdered Montmorency tart cherry supplement capsule (66 mg anthocyanin) or placebo (rice flour), taken once daily for 10 days, including the day of the race and up to 48 h post-run after breakfast	Half-marathon run	Algometer with a graphic pain rating scale	Change in soreness perception from pre-run in vastus medalis was significantly smaller in the placebo group compared to the tart cherry group over 48-h (*P* = 0.035); no differences in soreness perception in other locations of quadriceps (*P* > 0.05).
McCormick 2016 (15)	Cross-over trial	Nine highly-trained male Water Polo players, age 18.6 ± 1.4 years	90 ml/d Montmorency tart cherry concentrate (9.117 mg/ml anthocyanin) diluted with water or placebo (fruit cordials) for 6 days (exercise on day 6)	The exercise protocol included swimming-based tests: in-water vertical jump test, 10 m sprint test, repeat sprint test, and Water Polo Intermittent Shuttle Test.	VAS	No treatment effect on DOMS over 6 days (*P* > 0.05)
Bell 2015 (32)	Parallel trial	16 trained male cyclists, age 30 ± 8 years	Montmorency cherry concentrate (30 ml with 9.2 mg/ml anthocyanin) or placebo (mixed berry cordial), mixed with 100 ml water, twice a day, for 8 consecutive days (4 days pre-trial, on the day of, and 3 days post-trial)	A 109-min cycling trial designed to replicate road race demands	VAS	No treatment effect on DOMS over 72 h post-exercise (*P* > 0.05).
Levers 2015 (31)	Parallel trial	23 resistance-trained males, age 20.9 ± 2.6 years	480 mg freeze-dried powdered Montmorency tart cherry supplement capsule (40 mg anthocyanin per 290 mg) or placebo (rice flour), taken once daily for 10 days (7 days before exercise, on the day of exercise, and for 2 days after exercise)	10 sets of 10 repetitions at 70% of 1-RM back squat exercise	Algometer with a graphic pain rating scale	Muscle soreness perception was significantly lower in the tart cherry group compared to the placebo group for vastus lateralis (*P* = 0.024) with ratings increased by 55–170% in placebo and 35–104% in tart cherry group over the recovery. DOMS also tended to be lower in the tart cherry group for vastus medialis muscles (*P* = 0.10); soreness increased by 28–83% in placebo but ranged from 6% decrease to 58% increase in tart cherry group over the recovery.
Kastello 2014 (33)	Cross-over trial	14 untrained adults, age: 21.3 ± 2.8 years	Tart cherry supplement with other ingredients (containing 100 mg anthocyanins, 20 mg flavones, and 30 mg tannins, melatonin, and two flavonoids isoquercitrin and quercitrin) or placebo (cooking oil with food color), taken twice a day for 16 days prior to and 3 days	Five sets of 10 maximal eccentric arm extensions, totaling 50 maximal contractions	VAS and algometer	Significantly less pain in both objective and subjective measures in the tart cherry group compared to the placebo group (objective measure: time by treatment *P* = 0.03, at 48 h mean VAS was 4.1 vs. 4.9 for tart cherry vs.
			following eccentric exercise protocol			placebo; objective measure: time by treatment *P* = 0.04; at 12 h after exercise, mean force was 1.5 vs. 1.4 kg for tart cherry vs. placebo).
Bowtell 2011 (34)	Cross-over trial	10 well-trained male athletes participating in high-intensity intermittent sports with regular resistance training, age 27.8 ± 1.6 years	30 ml of Montmorency cherry juice concentrate (9.117 mg/ml anthocyanin) or placebo (synthetically derived fruit concentrate) twice per day for 10 days (7 days before exercise and 48 h after exercise)	10 sets of 10 single-leg knee extensions performed at 80% of 1-RM.	Algometer	No treatment effect on pressure pain threshold over 48 h after exercise in three muscles (*P* > 0.05)
Kuehl 2010 (41)	Parallel trial	54 healthy runners, age 35.8± 9.6 years	Montmorency tart cherry juice (355 ml with ≥600 mg phenolic compounds and ≥40 mg anthocyanins, twice daily) or placebo (fruit punch soft drink mix with water), for 7 days prior to the running event and on the day of the race.	Running in the Oregon Hood to Coast relay race (three separate segments over a 24-h period).	VAS	There was a significantly smaller increase in pain in the cherry juice group compared to the placebo group after the race (pain score was 22.6 ± 12.6 vs. 45.3 ± 20.5 mm for tart cherry vs. placebo, *P* < 0.001).
Howatson 2010 (23)	Parallel trial	20 recreational marathon runners, age 37 ± 13 for tart cherry juice group and 38 ± 5 for placebo group	Montmorency tart cherry juice (8 oz containing ≥600 mg phenolic compounds and ≥40 mg anthocyanins) or placebo (fruit-flavored water), twice a day, for 5 days before, the day of, and 48 h after a marathon run	A full marathon run	VAS	No treatment effect on DOMS over 48 h after the race; at 48 h, pain score was 58 ± 39 vs. 46 ± 28 mm for tart cherry vs. placebo, *P* > 0.05
Connolly 2006 (35)	Cross-over trial	14 male college students, age 22 ± 4 years	12 fl oz of a Montmorency tart cherry juice blend (containing at least 600 mg phenolic compounds and at least 40 mg anthocyanins) or placebo (black berry Kool-Aid drink with water), twice a day for eight consecutive days (exercise on day 4)	Eccentric elbow flexion contractions (two sets of 20 maximal contractions)	Likert scale	There was significantly less pain in elbow flexors post-exercise when tart cherry was consumed than placebo over 4 days (treatment by time interaction *P* = 0.017). Pain peaked at 24 h (2.4 ± 0.7) in the tart cherry trial then declined, but pain continued to increase to peak at 48 h in the placebo trial (4.5 ± 1.7).

## 4 Discussion

To our knowledge, this is the first review of Montmorency tart cherry and its effect on sport-related outcomes. This scoping review identified 28 randomized controlled trials that examined the effect of Montmorency tart cherry on athletic performance, post-exercise acute recovery of muscle strength, or DOMS in healthy adults. Most studies used US-sourced Montmorency tart cherry. Nonetheless, there were inconsistent results across studies, likely due to heterogeneity in study design. For example, there was no consensus on the dose or the number of days for supplementation, as well as when Montmorency tart cherry was consumed before the exercise. There was also a mixture of field studies and laboratory studies, as well as a mixture of studies in professional athletes and studies in recreationally active adults.

### 4.1 Potential mechanisms

Four of 10 studies included in this review reported a significant improvement in athletic performance with Montmorency tart cherry consumption based on time to complete exercise or time to exhaustion. A previous meta-analysis also revealed a significant effect of tart cherry on endurance exercise performance, although it was not exclusively based on findings from studies with Montmorency tart cherries (6). The mechanism for the enhanced performance may be related to increased muscle oxygenation and tissue oxygen saturation following tart cherry consumption, as reported in previous studies (17, 19). Anthocyanin has been reported to enhance nitric oxide production via activation of endothelial nitric oxide synthase (42), which can improve blood flow, leading to increased delivery of oxygen to muscles during exercise (43).

A moderate number of studies also revealed significant effects on post-exercise recovery of muscle strength and reductions in DOMS, further supporting conclusion from a meta-analysis that tart cherry supplementation can reduce DOMS and help with recovery of muscle strength (8). The effects of Montmorency tart cherries on post-exercise recovery are likely explained by their antioxidant and anti-inflammatory phytochemicals. Phenolic compounds and flavonoids such as anthocyanin in tart cherries can reduce both oxidative stress and inflammation *in vivo* (44, 45). This is also supported by findings from a meta-analysis reporting a significant reduction in anti-inflammatory markers such as interleukin-6 and C reactive protein following tart cherry supplementation (8). In addition, mechanisms for the effect on DOMS may be also through improved pain sensitivity as anthocyanin has been shown to dose-dependently reduce inflammation-induced hyperalgesia (46).

### 4.2 Limitations of existing studies

It should be noted that most of the studies included in this review had a limited sample size, had a relatively short intervention period, and included mostly young men in their study populations. The small sample size and short intervention period may have contributed to some of the non-significant findings due to lack of statistical power. Similarly, generalizability of the findings may have been limited given most studies were conducted with young male athletes. Moreover, majority of studies did not pre-register their clinical trial and did not specify a primary outcome. Lastly, pharmacokinetics and pharmacodynamics of anthocyanin after Montmorency tart cherry consumption has received little attention, until a recent study that examined such outcomes in patients with gout and reported large variations in those parameters (47). Further understanding of individualized metabolite profiles following Montmorency tart cherry consumption may offer insights for personalized nutrition recommendations for optimal benefit on exercise and recovery, as well as to identify the best timing for consumption prior to exercise in acute settings.

### 4.3 Strengths and limitations of the review

The present scoping review evaluated the latest evidence in the published literature to address the research question. Furthermore, the initial search term did not include any terms related to sports or exercise, thereby reducing the risk of missing relevant literature. However, there are some limitations. First, although relevant references from review articles were manually checked, only one database was used for the initial literature search, and the scoping review was not prospectively registered. Second, only English literature was assessed, and this may have resulted in a bias toward inclusion of studies with US or Canadian Montmorency tart cherries. Third, the review only included direct outcomes related to athletic performance, as well as muscle strength recovery and DOMS; additional outcomes such as biomarkers were not included. Lastly, due to the nature of a scoping review, risk of bias of included studies was not quantitatively assessed, nor was there a quantitative assessment on effect sizes.

### 4.4 Future directions

In February 2026, the International Society of Sports Nutrition published its official position on the role of dietary antioxidants in exercise and sport (48). Montmorency tart cherry is one of the few common sources of antioxidant that was considered to have “moderate” or “high” level of evidence to support exercise and sport (48). Nevertheless, several further directions could still be considered. First, clinical studies with a larger sample size and a longer intervention are desired. Second, study populations could be expanded to include adolescents and older adults. Third, the dose-response relationship of Montmorency tart cherry and outcomes should be examined with multiple doses to identify the most effective dose. Fourth, the effect of phenolic compounds in the background diets needs to be addressed to better isolate the effect of Montmorency tart cherry consumption. Lastly, a systematic review and meta-analysis is warranted to further assess and quantify the totality of evidence and effect size in the published literature.

## 5 Conclusions

Despite inconsistent findings, there was preliminary evidence from published studies to support the potential beneficial role of Montmorency tart cherry on certain exercise-related outcomes in healthy adults.

Future studies are warranted to examine the optimal dose and timing of Montmorency tart cherry intake and to evaluate the effect of long-term supplementation in more diverse study populations.
